# Successful Treatment of Unscheduled Uterine Bleeding During Transdermal Menopausal Hormone Therapy Combined With Bazedoxifene

**DOI:** 10.7759/cureus.81582

**Published:** 2025-04-01

**Authors:** Tiger Koike, Koji Koike, Tomomi Shiga, Motoki Takenaka, Tatsuro Furui

**Affiliations:** 1 Gynecology, Koike Ladies Clinic, Osaka, JPN; 2 Obstetrics and Gynecology, Gifu University Hospital, Gifu, JPN

**Keywords:** abnormal uterine bleeding, bazedoxifene, menopausal hormone therapy, tissue-selective estrogen complex, transdermal 17-beta estradiol

## Abstract

Abnormal uterine bleeding and withdrawal bleeding are noted as crucial causal factors for dropout of postmenopausal women during menopausal hormone therapy (MHT). We report the potential for treatment of unscheduled uterine bleeding during transdermal MHT by switching to transdermal 17-beta estradiol (TDE_2_) and bazedoxifene acetate (BZA) (TDE_2_/BZA) and evaluate the side effects of this treatment on the recurrence of climacteric symptoms in the five reported patients. Five postmenopausal women were treated with estrogen-progestin therapy (EPT) which included TDE_2_ and progestin formulation for MHT. The progestin formulation was replaced with BZA, a selective estrogen receptor modulator, due to unscheduled uterine bleeding. Four of five postmenopausal women were evaluated in terms of estrogen dynamics and recurrence of climacteric symptoms. In all cases examined in this report, unscheduled uterine bleeding resolved within one month. In two of four patients in whom climacteric symptoms recurred, their serum estradiol levels were surprisingly elevated three months after the start of TDE_2_/BZA. This is the first report, to our knowledge, of the successful treatment of unscheduled uterine bleeding by TDE_2_/BZA during MHT and a suggested correlation between estrogen dynamics and concomitant recurrence of side effects caused by TDE_2_/BZA.

## Introduction

Menopausal hormone therapy (MHT) is considered the gold standard for management of climacteric symptoms. The route of administration of estrogen and progestin is oral, transdermal, vaginal, and intrauterine devices. Estrogen therapy (ET) can be administered for women without a uterus; otherwise, estrogen-progestin therapy (EPT) is recommended for women with a uterus in principle to protect the endometrium. With EPT, progestin can be administered either sequentially or continuously. However, the problem in performing EPT is uterine bleeding because uterine bleeding impairs quality of life and is a crucial cause of dropout by patients treated with MHT [1]. Withdrawal bleeding is caused by sequential EPT, and abnormal uterine bleeding (AUB) sometimes occurs with continuous EPT. Duavee (Pfizer), approved by the U.S. Food and Drug Administration in 2013, is a first drug of the new MHT method called tissue-selective estrogen complex (TSEC) therapy, which combines a third-generation selective estrogen receptor modulator, bazedoxifene (BZA), with conjugated equine estrogen (CEE) instead of progestin for postmenopausal women with or without a uterus [2]. TSEC is intended to treat postmenopausal osteoporosis and vasomotor symptoms (VMS) [2], and one of its unique additional effects is the suppression of uterine bleeding [3]. However, there have been reports on TSEC using oral estrogens, but none using transdermal estrogens. As for estrogen, it has been reported that oral administration including CEE increases the risk of thrombosis due to hepatic first-pass effect, whereas the use of transdermal 17-beta estradiol (TDE_2_) does not increase [4].

This is the first report, to our knowledge, of combination therapy including TDE_2_ and BZA (TDE_2_/BZA) to treat unscheduled uterine bleeding of postmenopausal women during MHT.

## Case presentation

Case 1

A 50-year-old woman was diagnosed as having osteoporosis with a bone mineral density of 63% of the young adult mean at the right femoral neck by dual-energy X-ray absorptiometry. The patient presented to our university hospital with torsion of a left ovarian mature cystic teratoma 10 years before, for which the patient was admitted for left salpingooophorectomy. Two months after the surgery, the patient developed a right ovarian abscess and was re-admitted for a right salpingooophorectomy. Thereafter, the patient was started on MHT with transdermal norethisterone and TDE_2_ to treat surgical menopause. The patient had complained of AUB since the start of MHT, but MHT was continued at her request. Cytology of the endometrium demonstrated no abnormal findings. There were no polyps or other benign lesions, and the endometrium was less than 2 mm thick with no findings suggesting endometrial hyperplasia. Progestin was replaced by BZA, and the estrogen formulation was switched to TDE_2_ gel. In this case, the estradiol gel was started at a lower dose (0.9 g of E_2_ designed to release 0.54 mg per day of E_2_ continuously on application) at the beginning of treatment.

The patient’s AUB was resolved within one month after the start of TDE_2_/BZA. However, mild VMS were observed. Three months after the switch of treatment, VMS became severe and other climacteric symptoms were observed. The manifested symptoms were shoulder stiffness, headache, and vertigo. Therefore, the patient’s E_2_ dose was increased to the standard dose (1.8 g of E_2_ designed to release 1.08 mg per day of E_2_ continuously on application). As a result, VMS and other climacteric symptoms were resolved within two months. During treatment, no adverse events were observed except for climacteric symptoms, and treatment is continuing without AUB. Endometrial cytology was performed every 12 months, and ultrasonography was performed every six months. The thickness of the endometrium was kept less than 2 mm with no findings suggesting endometrial hyperplasia during TDE_2_/BZA.

Case 2

**Figure 1:**
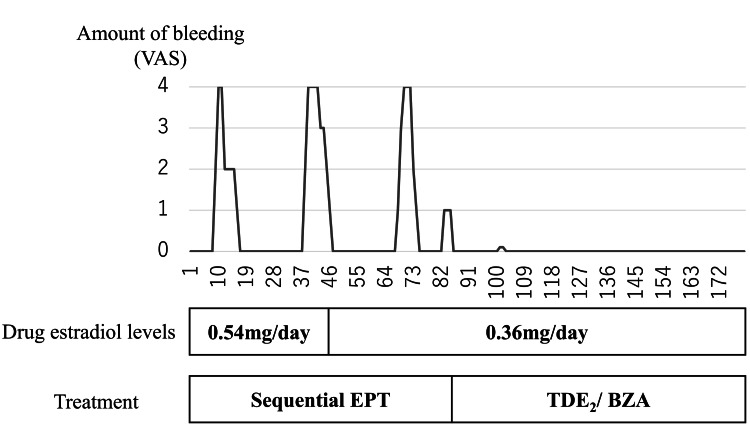
The amount of bleeding during the three months before and after switching of treatment in Case 2 The maximum amount of menstrual blood volume for the patient’s young age (20–39 years old) was defined as Score three on the visual analogue scale (VAS). EPT, estrogen-progestin therapy; TDE_2_/BZA, transdermal 17-beta estradiol/bazedoxifene acetate.

A 54-year-old postmenopausal woman with a history of climacteric symptoms presented to our clinic for treatment by sequential MHT. Transdermal E_2_ skin patch (containing 0.72 mg of E_2_ designed to release 50 µg per day of E_2_ continuously on application) and medroxyprogesterone acetate (MPA) were selected. After the patient complained of breast discomfort, the E_2_ dose was decreased from 0.72 mg/day to 0.54 mg/day. Three years later, she complained of increasing withdrawal bleeding, and the E_2_ dose was decreased again from 0.54 mg/day to 0.36 mg/day. One month thereafter, the patient complained of increasing withdrawal bleeding again which sometimes caused AUB. The MPA was replaced at this time with BZA. The large amount of withdrawal bleeding and AUB were resolved within one month (Figure 1). Treatment is continuing without any side effects. Endometrial cytology and ultrasonography were performed every six months from the start of MHT. The thickness of the endometrium tends to decrease with no findings suggesting endometrial hyperplasia during TDE_2_/BZA.

Case 3

A 49-year-old perimenopausal woman with a history of difference in the length of consecutive menstrual periods presented to our clinic for treatment by sequential MHT. Transdermal E_2_ skin patch (containing 0.72 mg of E_2_ designed to release 50 µg per day of E_2_ continuously on application) and MPA were selected. The patient went through menopause at 50 years old. One year after menopause, the patient complained of increasing withdrawal bleeding, and the MPA was replaced with BZA. The large amount of withdrawal bleeding was resolved within one month, but three months later, the patient began to complain of sweating and hot flashes. The clinician added a traditional Japanese medicine (Kampo), Boiogito, which is well-known to heal severe sweating and the symptoms were resolved in one month. Endometrial cytology and ultrasonography were performed every six months from the start of MHT. The thickness of the endometrium tends to decrease with no findings suggesting endometrial hyperplasia during TDE_2_/BZA.

Case 4

A 55-year-old postmenopausal woman with a history of climacteric symptoms presented to our clinic for treatment by sequential MHT. Transdermal E_2_ skin patch (containing 0.72 mg of E_2_ designed to release 50 µg per day of E_2_ continuously on application) and dydrogesterone (DYD) were selected. Ten months later, the patient complained of increasing withdrawal bleeding, and DYD was replaced with BZA. The withdrawal bleeding was resolved within one month. Treatment is continuing without any side effects. Endometrial cytology and ultrasonography were performed every six months from the start of MHT. The thickness of the endometrium tends to decrease with no findings suggesting endometrial hyperplasia during TDE_2_/BZA.

Case 5

A 61-year-old postmenopausal woman, with a history of climacteric symptoms and treated for six years, presented to our clinic for restart treatment by sequential MHT. Transdermal E_2_ gel (1.8 g of E_2_ designed to release 1.08 mg per day of E_2_ continuously on application) and DYD were selected. Two years later, the patient complained of continuous AUB. The E_2_ dose was decreased from 1.08 mg/day to 0.81 mg/day. However, general fatigue was actualized. Thereafter, the estradiol dose was controlled at 1.08 mg/day in summer and 0.81 mg/day in winter. However, six years later, the patient complained of a floating sensation and increasing AUB. The E_2_ dose was fixed to 1.08 mg/day, and DYD was replaced with BZA. The continuous AUB was resolved within one month, but three months later, the patient began to complain of hot flashes and joint pain. The patient wanted to switch TDE_2_/BZA to sequential EPT, which the patient had previously done. BZA was replaced with DYD. Four months later, the patient complained about the recurrence of increasing AUB. The patient wished to change again EPT to TDE_2_/BZA. Endometrial cytology and ultrasonography were performed every six months from the start of MHT. The thickness of the endometrium tends to decrease with no findings suggesting endometrial hyperplasia during TDE_2_/BZA.

Correlation between recurrence of climacteric symptoms and estrogen dynamics in four cases

**Figure 2:**
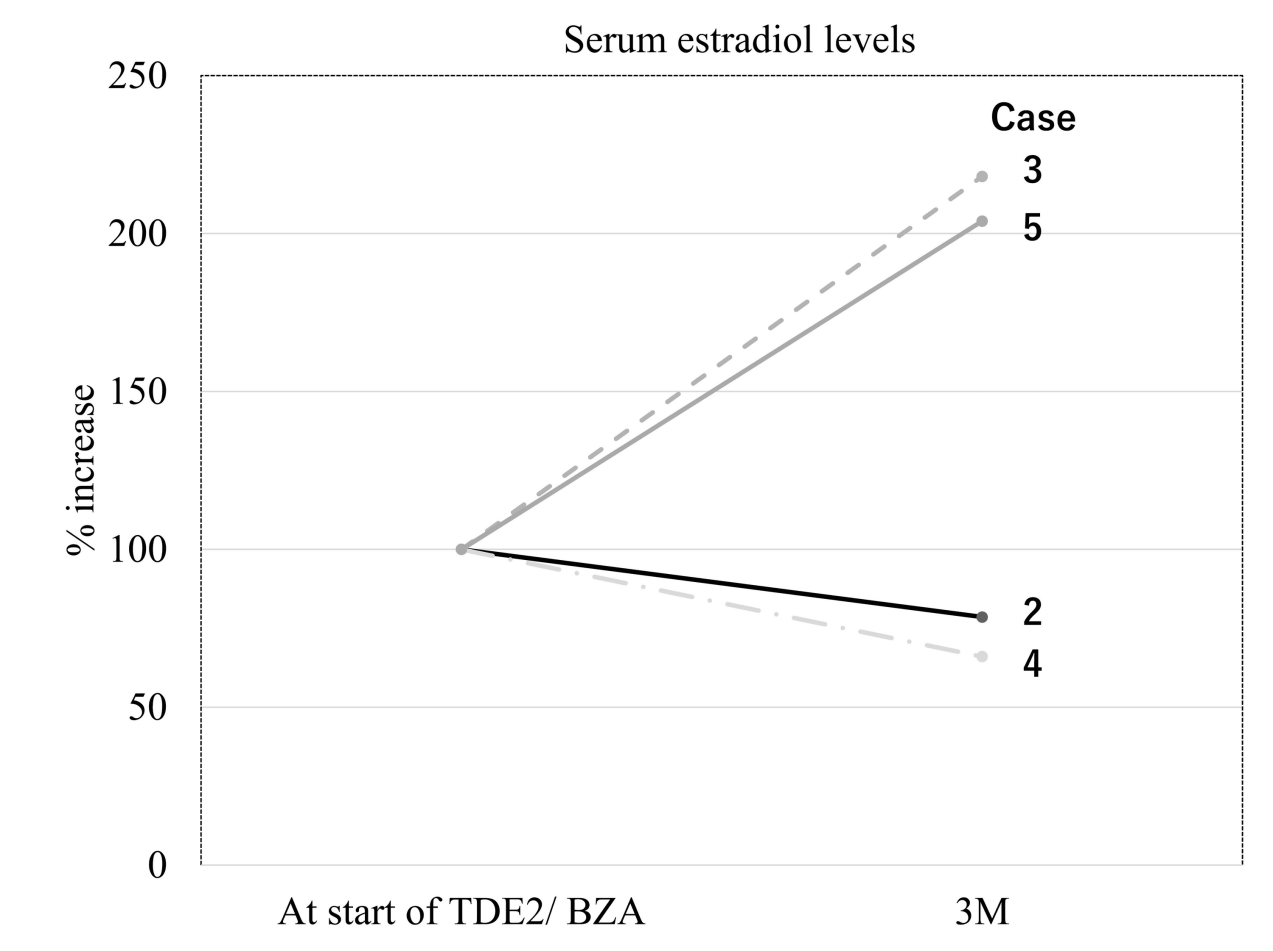
Changes in serum estradiol levels before and after switching of treatment TDE_2_/BZA, transdermal 17-beta estradiol/bazedoxifene acetate; 3M, three months after the switch of treatment.

Four postmenopausal women, including cases 2, 3, 4, and 5 with a history of climacteric symptoms who presented to our clinic for treatment with MHT were switched from sequential EPT to TDE_2_/BZA and were evaluated for estrogen dynamics and recurrence of climacteric symptoms. Case 1 was the first case of TDE_2_/BZA we experienced but was excluded because the laboratory system was different due to the use of different facilities, the timing of blood collection was different, and the E_2_ dose was changed during TDE_2_/BZA therapy. These four patients presented with a large amount of withdrawal bleeding or AUB that occurred during MHT (sequential EPT), and their bleeding was resolved within one month. However, the recurrence of climacteric symptoms was noted in two of the four cases almost three months after switching treatment. Serum E_2_ and follicle-stimulating hormone (FSH) levels were measured, and although FSH was elevated in all patients, E_2_ was elevated only in the patients with recurrence of climacteric symptoms (Table 1, Figure 2). Recurrent climacteric symptoms included hot flashes, sweating, and joint pain. 

**Table 1:** Characteristics of the cases In case 1, blood samples were collected at one and five months after the switch of treatment. FSH, follicle-stimulating hormone, TDE_2_/BZA, transdermal 17-beta estradiol/bazedoxifene acetate; 3M, three months after the switch of treatment. The reference range of FSH for post-menopausal women is 157.79 mIU/mL or less. The reference range of E_2_ for post-menopausal women is less than 20 pg/mL.

Case	Age	Serum hormone levels			Recurrence of climacteric symptoms		Hemoglobin (g/dL) (Reference range: 11.6- 14.8)		D-Dimer (μg/mL) (Reference range: <1.0)	
		Hormone	At the start of TDE_2_/ BZA	3M	+/-	Symptom	At the start of TDE_2_/ BZA	3M	At the start of TDE_2_/ BZA	3M
Case 1	58	FSH (mlU/mL)	62.1 (1M)	58.6 (5M)	+	Hot flash, sweating, shoulder stiffness, headache, vertigo	12.8 (1M)	No data	<0.5 (1M)	No data
		E_2_ (pg/mL)	26 (1M)	212 (5M)						
Case 2	58	FSH (mlU/mL)	31.7	38.6	-		11	12.1	<0.5	<0.5
		E_2_ (pg/mL)	47.6	37.4						
Case 3	51	FSH (mlU/mL)	69.0	111.3	+	Hot flash, sweating	9.5	12	<0.5	<0.5
		E_2_ (pg/mL)	126.5	275.9						
Case 4	56	FSH (mlU/mL)	19.2	78	-		9.1	10.6	0.7	<0.5
		E_2_ (pg/mL)	70.4	46.5						
Case 5	69	FSH (mlU/mL)	48.6	53.1	+	Hot flash, joint pain	11.1	13.2	0.9	<0.5
		E_2_ (pg/mL)	58.3	118.9						

## Discussion

TSEC is often focused on improving postmenopausal osteoporosis and VMS, which are its indications [2]. However, many other benefits have been reported. Its stimulating effects on the mammary gland and endometrium have been reported to be equivalent to those of placebo [5], and its effectiveness in vascular protection is reported in many cases [6]. In particular, it should also be noted that TSEC for postmenopausal women is associated with less uterine bleeding [7]. Although both CEE and BZA carry the risk of thrombosis as a side effect [4,8], the phase three trial for Duavee did not show an increased risk of thrombosis after a short period of two years of oral administration [2]. The SMART5 trial shows that the incidences of venous thromboembolic events and cardiovascular events in postmenopausal women who were treated with CEE 0.45 mg/BZA 20 mg are similar to those observed with placebo in 12 months [9]. One of the studies of TSEC with oral E_2_ and raloxifene was stopped early because it showed a significant increase in endometrial hyperplasia [10]. However, this may be due to raloxifene's weaker antagonistic effect on the endometrium compared to bazedoxifene [11]. No adverse events including endometrial hyperplasia have been reported in previous studies using oral E_2_ and BZA in 12 months [12]. Therefore, we selected BZA for SERM and transdermal 17-beta estradiol (TDE_2_) for estrogen, because it has been reported that the use of TDE_2 _does not increase the risk of thrombosis [4] and may be a safe choice for long-term TSEC therapy.

In case 1, E_2_ gel was started at a lower dose (half the standard dose) at the beginning of TDE_2_/BZA therapy. However, VMS were observed at lower doses, but the symptoms disappeared after increasing to the standard dose. In the SMART2 trial, CEE 0.3 mg (half the standard dose) did not control VMS with BZA 20 mg [13]. This is also consistent with the course of our present case. Recently, a clinical trial on bone metabolism rotation using TSEC at a standard dose (1 mg) of oral micronized E_2_ and BZA was reported, and no apparent adverse events were reported [12]. In general, 1 mg of micronized E_2_ is considered equivalent to 0.72 mg delivered by E_2_ patch and 1.8 g of E_2_ gel. Therefore, the standard dose of TDE_2_ in this treatment should be 0.72 mg of E_2_ via patch and 1.8 g of E_2_ gel. Case 1 was the first TDE_2_/BZA case we experienced. Therefore, based on the course of case 1, we checked blood samples for management purposes before the start of the study and in the third month after menopausal symptoms became apparent in subsequent cases. Recurrence of menopausal symptoms was also checked by filling out a VAS for each symptom every month.

In cases 2 and 4, TDE_2_/BZA was effective for the treatment of a large amount of withdrawal bleeding and AUB without causing side effects. In cases 3 and 5, however, recurrences of climacteric symptoms were observed, although the large amount of withdrawal bleeding was resolved.

Therefore, four postmenopausal women, including cases 2, 3, 4, and 5, were evaluated in terms of estrogen dynamics and recurrence of climacteric symptoms. We found that serum E_2_ levels were elevated only in the cases showing recurrence of climacteric symptoms. The possibility of climacteric symptoms appearing when changing from EPT to TSEC has been reported [3], which is consistent with the recurrence of climacteric symptoms in our reviewed cases. In that report, a low dose of CEE for TSEC was cited as a cause of the appearance of climacteric symptoms. We speculate that in cases of recurrent climacteric symptoms, such as those we reviewed, the competition between BZA and E_2_ on estrogen receptor alpha (ERα) located on the KNDy neuron results in inadequate binding of E_2_ to ERα. As a result of the decreased tone of estrogen signaling in the KNDy neuron, which may have a key role in VMS [14], clinical recurrence of climacteric symptoms might be observed. An additional interesting finding is that an increase in the administered E_2_ dose may reverse recurrent climacteric symptoms caused by TDE_2_/BZA, as shown in case 1. This case suggested that increasing the ratio of blood E_2_/BZA could overcome the recurrent climacteric symptoms and also indicated the competitive inhibition between ER and BZA during TDE_2_/BZA therapy. TDE_2_ has been reported not to increase the risk of thrombosis even at double the standard dose [4]. However, high-dose TDE_2_ has also been reported to significantly increase the risk of stroke [15], and long-term administration of high-dose TDE_2_ may be undesirable. Therefore, other treatment modalities may be considered in combination to treat climacteric symptoms as shown in case 3. The recently developed neurokinin 3 receptor antagonist, fezolinetant [16], may also be a possible candidate.

Presently, however, it is unknown whether a correlation exists between recurrent climacteric symptoms and elevated levels of serum E_2_. The serum E_2_ detected is exogenous in origin as the cases targeted are postmenopausal women. It has been found that p450 (SYP3A4), which is involved in E_2_ metabolism, is not involved in BZA metabolism [17]. Therefore, it is not likely that an increase in serum E_2_ levels may be due to inhibition of the E_2_ metabolic process. We thus speculate that E_2_ and BZA compete for systemic distribution of ERs, and E_2 _that fails to bind to ERs may be detected as elevated levels of serum E_2_. It has been reported during TSEC that expressions of ERs increase or decrease in a tissue-specific manner [18-20]. To our knowledge, the present report is the first to discuss estrogen dynamics during TDE_2_/BZA treatment in postmenopausal women. Currently, it is unclear which factors are responsible for these different serum E_2 _levels between the cases in which climacteric symptoms recurred and the other cases. We also observe that some of the other cases by the same treatment show a concomitant increase of serum estradiol levels with recurrence of climacteric symptoms (data not shown), suggesting that this phenomenon is not rare. Further study is necessary to clarify the discrepancy between concomitant elevated serum levels of E_2_ and the recurrence of climacteric symptoms.

## Conclusions

This is the first report of the successful treatment of unscheduled uterine bleeding by TDE_2_/BZA during MHT. It is suggested that TDE_2_/BZA is an effective treatment for controlling unscheduled uterine bleeding as well as conventional TSEC and may be an optional method to treat this condition. In patients whose climacteric symptoms recurred, their serum E_2_ levels were surprisingly elevated three months after the start of TDE_2_/BZA, while in other patients without recurrent menopausal symptoms, serum E_2_ levels did not change. This finding suggests the presence of a correlation between increasing serum E_2_ level and concomitant recurrence of climacteric symptoms after switching to TDE_2_/BZA therapy. However, further clinical study in increasing numbers is necessary to clarify this finding.
